# The Effect of Highly Virulent PRRSV-2 (L1C.5) Infection on Calcium Homeostasis and Bone Changes in Pigs Fed with Various Levels of Dietary Vitamin D

**DOI:** 10.3390/v18070711

**Published:** 2026-06-27

**Authors:** Panchan Sitthicharoenchai, Kelly Grace Keen, Veeraya Bamrung, Chareerut Phruksaniyom, Sara Hough, Eric van Heugten, Devorah Stowe, Jianqiang Zhang, Michael C. Rahe

**Affiliations:** 1 Department of Population Health and Pathobiology, College of Veterinary Medicine, North Carolina State University, Raleigh, NC 27607, USA; kgkeen@ncsu.edu (K.G.K.); veerayab@iastate.edu (V.B.); cphruks@ncsu.edu (C.P.); damarks@ncsu.edu (D.S.); 2 Department of Veterinary Diagnostic and Production Animal Medicine, College of Veterinary Medicine, Iowa State University, Ames, IA 50011, USA; jqzhang@iastate.edu; 3 dsm-firmenich, Plainsboro, NJ 08536, USA; sara.hough@dsm.com; 4 Department of Animal Science, College of Agriculture and Life Sciences, North Carolina State University, Raleigh, NC 27607, USA; eric_vanheugten@ncsu.edu

**Keywords:** bone marrow, myelonecrosis, PRRSV, swine, vitamin D

## Abstract

Porcine reproductive and respiratory syndrome virus (PRRSV) is a major cause of disease in swine production, resulting in a high economic impact that has been exacerbated by the recent North American outbreaks of highly virulent PRRSV-2 strain (L1C.5). However, there is limited knowledge about how underlying systemic infections, particularly with this emergent PRRSV strain, affect calcium regulation and bone in pigs fed varying levels of vitamin D. To address this, the goal of this study was to determine the effect of different dietary vitamin D levels and PRRSV infection on calcium regulation and phenotypic changes in bone. Three-week-old pigs (*n* = 42) were assigned to four treatment groups: marginal dietary vitamin D3 (200 IU/kg) + PRRSV (*n* = 12), industry standard dietary vitamin D3 (1500 IU/kg) + PRRSV (*n* = 12), industry standard dietary vitamin D3 (1500 IU/kg) supplemented with 25-hydroxy-vitamin D3 (25-OH D3) (2000 IU/kg) + PRRSV (*n* = 12), or marginal dietary vitamin D3 without PRRSV inoculation (200 IU/kg) (control, *n* = 6). Following 26 days of dietary acclimation, assigned treatment groups were inoculated with a PRRSV-2 L1C.5 isolate. Blood samples were collected to evaluate the ionized calcium, 25-OH D3, calcium, phosphorus, and parathyroid hormone levels. The 2nd and 10th ribs were collected at 14 days post challenge for bone ash and density analysis, as well as examination of microscopic changes and scoring of the physis. High mortality was noted in all pigs infected with the virus, regardless of the vitamin D diet. Additionally, a significant depletion of serum calcium was observed at 7 DPC in infected animals, suggesting a high calcium demand at early stages of PRRSV infection. No significant differences in serum calcium, phosphorus, or ionized calcium concentrations were observed between dietary groups during the first 14 days of PRRSV-2 L1C infection. In pigs that succumbed to PRRSV at the early stage of infection, microscopic lesions of multifocal myelonecrosis were noted. This study provides the first report of microscopic changes of bone marrow necrosis and inflammation associated with PRRSV infection and demonstrates calcium dysregulation at the early stage of infection by this highly virulent PRRSV strain.

## 1. Introduction

Porcine reproductive and respiratory syndrome (PRRS) is one of the most economically important diseases in swine production globally. It remains endemic in many regions, including the United States [1,2]. The clinical manifestations of PRRS include respiratory disease, immunosuppression, reproductive failure, and occasionally sudden death. The causative agent, PRRS virus (PRRSV), is an enveloped, single-stranded RNA virus in the family Arteriviridae. This genetically diverse virus is categorized into 2 species, PRRSV-1 (*Betaarterivirus europensis*) and PRRSV-2 (*Betaarterivirus americense*), with multiple lineages and sub-lineages in each type based on the open reading frame 5 (ORF5) sequences [2,3,4]. The recent outbreaks of a highly virulent strain of PRRSV-2 L1C.5 emerged in North America at the beginning of October 2020, causing high mortality and morbidity in pigs in all age groups, particularly in nursery pigs [5,6,7,8]. PRRSV naïve pigs experimentally infected with PRRSV-2 L1C.5 showed severe clinical signs of coughing, anorexia, lethargy, and fever, with over 75% mortality by 14 days post-challenge (DPC), comparable to the pigs infected with highly pathogenic PRRSV reported in Asia [6,8]. Interstitial pneumonia with aggregates of necrotic macrophages filling alveolar spaces is the key microscopic finding with PRRSV infection. Other PRRSV-associated lesions include lymphoid depletion, nephritis, myocarditis, and meningoencephalitis [9]. Bone marrow hypoplasia, increased ratio of myeloid to erythroid precursors, and apoptosis of hematopoietic precursor cells have been characterized in the bone marrow of pigs with PRRSV infection [10,11,12].

Vitamin D is critical for regulating calcium and phosphorus absorption, which is essential for musculoskeletal development. During infection, the role of vitamin D shifts from bone maintenance to immune modulation via the vitamin D receptor signaling of immune cells [13]. Furthermore, certain viral infections have been reported to cause hypocalcemia and suppression of parathyroid hormone [14,15,16]. Commercial pigs are faced with the challenge of lower de novo synthesis of cholecalciferol due to limited exposure to sunlight and require supplementation of exogenous vitamin D through their diet. A recent survey on vitamin D supplementation in nursery-to-finishing pig diets in the US commercial swine industry showed that pigs are commonly fed vitamin D at concentrations ranging from 6.0 to 11.8 times the requirement suggested by the National Research Council (NRC) (2012) [17,18]. Similar levels of supplementation are noted in nursery and finisher diets in Brazil [19] as well as in finisher pigs in China [20]. Regardless, commercial grower pigs may still have marginal levels of serum 25(OH) D3 even when fed vitamin D levels above the NRC-suggested requirements [21,22]. When compounded by physiological stress during an infectious disease outbreak, this can result in hypovitaminosis D manifesting clinically as lameness and metabolic bone disease [22]. In PRRSV endemic areas, the synergy between viral infection and vitamin D and calcium dysregulation is a significant concern.

To better understand how this newly emerging, highly virulent PRRSV-2 strain affects calcium regulation and bone development and whether vitamin D can help mitigate these effects, this study investigated bone lesions and calcium regulatory biomarkers in infected pigs fed different levels of vitamin D. This research addresses the limited information regarding the relationship between PRRSV infection and alterations in calcium homeostasis and bone health.

## 2. Materials and Methods

### 2.1. Animals

At the North Carolina State University Swine Education Unit, a total of 42 3-week-old, weaned commercial crossbred pigs (body weight of 6.14 kg ± 0.11) obtained from a PRRSV-negative multiplier were blocked by body weight, sex, and litter of origin and randomly assigned within blocks to one of four groups. Three of the groups were fed diets containing either marginal dietary vitamin D3 (200 IU/kg) (Marginal, n = 12), industry dietary vitamin D3 (1500 IU/kg diet) (Standard, n = 12), or industry dietary vitamin D3 supplemented with 25-OH D3 (HyD^®^, dsm-firmenich, Plainsboro, NJ, USA) (2000 IU/kg) (Supplemented, n = 12). These three groups were inoculated with PRRSV. The fourth group served as a negative control receiving the marginal dietary vitamin D3 diet without PRRSV inoculation (Control, n = 6). The pigs had ad libitum access to their respective treatment diets immediately after weaning for twenty-two days after weaning. This pre-challenge acclimation period was established based on previous studies to allow vitamin D supplementation to take full effect [23,24]. Following this period, all pigs were moved to the North Carolina State University College of Veterinary Medicine animal BSL-2 facility. Control pigs were housed in a separate building with separate ventilation and caretakers. A temperature monitoring microchip (Bio-Thermo^TM^, Destron Fearing, St. Paul, MN, USA) was implanted intramuscularly at the base of the ear for monitoring body temperature. Pigs were acclimated to their new environment for four days. Subsequently, pigs in the Marginal, Standard and Supplemented groups were inoculated via 1 mL intramuscular injection of 3 × 10^5^ TCID_50_/mL PRRSV-2 L1C.5 in the post-auricular area of the neck and 1 mL intranasally (0.5 mL in each nostril) inoculated using a mucosal atomization device fitted onto a syringe that dispenses particle sizes of 30–100 μm (Teleflex Medical, Morrisville, NC, USA). This resulted in a total infectious dose of 6 × 10^5^ TCID_50_ PRRSV-2 L1C.5 per pig. The Control group was sham inoculated with virus-negative media (Dulbecco’s modified Eagle’s medium) through the same routes and volume, as previously described with PRRSV inoculation. Blood samples were collected through venipuncture at −26, −19, −12, −5, 0, 7, and 14 days post-challenge (DPC). Serum was extracted from the blood and stored at −80 °C until further evaluation. Pigs were weighed at inoculation and study end to calculate average daily gain. One pig from the Supplemented group died during blood collection before the PRRSV challenge. All pigs, including Control pigs, received an intramuscular injection of a single dose of ceftiofur crystalline free acid (Excede^®^, Zoetis Inc., Kalamazoo, MI, USA) at 5 mg/kg body weight at 10 DPC to prevent secondary bacterial infection. At 14 DPC, all pigs were sedated with an intramuscular injection of TKX (0.02–0.04 mL/kg (Telazol [50 mg/mL Tiletamine and 50 mg/mL Zolazepam], Ketamine-50 mg/mL, Xylazine-50 mg/mL) and euthanized with an intravenous injection of pentobarbital sodium (1 mL/4.5 kg). Pigs that showed severe clinical signs associated with PRRS, including open-mouthed breathing or neurologic symptoms, were sedated and euthanized immediately. Postmortem examinations were performed on all pigs by board-certified veterinary anatomic pathologists. The North Carolina State University Institutional Animal Care and Use Committee approved the protocol used in this study (IACUC No. 23-422).

### 2.2. Diet Composition and Preparation

Pigs were fed two diet phases to accommodate the gradual transition from sow milk to solid feed and to meet their nutritional requirements. Diets were formulated to be consistent with industry practices and met or exceeded nutrient requirements suggested by the 2012 NRC [18]. The phase 1 nursery diets were fed for 14 days immediately after weaning, and phase 2 nursery diets were fed for the remainder of the study. For each diet phase, a basal mix containing all ingredients except vitamins was created. Subsequently, treatment diets were manufactured by adding one of three specific vitamin premixes that provided either 200 IU/kg of vitamin D3 (Marginal and Control), 1500 IU/kg of vitamin D3 (Standard), or 1500 IU/kg of vitamin D3 plus 2000 IU/kg 25-OH D3 (HyD^®^, dsm-firmenich, Plainsboro, NJ, USA) (Supplemented). The vitamin premixes were chemically analyzed for vitamin D and 25-OH D3 and found to be within the accepted levels of analytical and sampling error, confirming that the diets contained the expected levels of vitamin D and 25-OH D3. The premixes contained identical levels of all other supplemental vitamins. The detailed ingredient composition and nutritional value of the feed mixture are included in Supplementary Table S1.

### 2.3. PRRSV Detection Using Quantitative Reverse Transcription Polymerase Chain Reaction (RT-qPCR)

PRRSV detection using RT-qPCR on serum samples was performed as previously described by Robinson et al., 2018 [25]. Briefly, RNA was isolated from the serum using PureLink Viral RNA/DNA Mini Kit (Life Technologies, Carlsbad, CA, USA). Complementary cDNA was synthesized with High-Capacity cDNA Reverse Transcription Kit (Applied Biosystems, Carlsbad, CA, USA). The primer sequences targeting the 198 bp ORF6-7 fragment of PRRSV-2 included AAC CAC GCA TTT GTC GTC (forward primer) and reverse primer sequence TGG CAC AGC TGA TTG ACT GG (reverse primer). SYBR green PCR Master Mix (PerfeCTa SYBR Green FastMix, Low ROX, Quanta Biosciences, Beverly, MA, USA) was used as the reporting system. Reactions were run in a qTower3G thermal cycler (Analytik Jena, Upland, CA, USA), with activation at 95 °C for 1 min, 45 cycles of denaturation at 95 °C for 3 s and annealing/extension at 60 °C for 25 s, followed by a dissociation step at 70–90 °C.

### 2.4. Evaluation of Whole Blood Ionized Calcium, Serum Calcium and Phosphorus, and Parathyroid Hormone

Heparinized blood samples were collected and analyzed for ionized calcium and blood gas profile using the i-STAT Alinity and CG8+ cartridge according to the manufacturer’s instructions (Abbott, Princeton, NJ, USA). The adjusted ionized calcium was calculated to a pH of 7.4. Serum was submitted to North Carolina State University Veterinary Diagnostic Service for routine large animal serum chemistry analysis to evaluate the serum calcium and phosphorus levels using a chemistry analyzer (Roche Cobas C501, Roche, Rotkreuz, Switzerland). Measurement of parathyroid hormone (PTH) was performed at the Michigan State University Veterinary Diagnostic Laboratory using a chemiluminescent immunometric assay [26]. Serum 25-OH D3 was evaluated at the Heartland Assay LLC (Ames, IA, USA) using the high-performance liquid chromatography (HPLC) technique [27].

### 2.5. Bone Sample Collection and Analysis

The proximal femur and 2nd and 10th ribs were collected during necropsy at 14 DPC. Bone density and bone ash were determined from the 2nd ribs [22,28]. Briefly, the bone density was evaluated according to Archimedes’ principle by subtracting the dried bone weight in air from the weight under water. To determine the bone ash level, the bones were placed in a furnace at 600 °C for 24 h. The ash was weighed to calculate the percentage of ash relative to the dried bone weight. The 10th rib and the proximal femur were placed in 10% neutral-buffered formalin for at least 24 h and later moved into formic acid to decalcify the bone. The decalcified bones were processed using routine procedures, embedded in paraffin, sectioned at 5 μm, and stained with hematoxylin and eosin. The areas at the costochondral junction and the physeal region at the head of the femur were evaluated for the degree of failure of endochondral ossification (physeal score) and evidence of infarctions or fractures [22]. The physis grading consisted of the following scale: (0) no histologic findings of significance, (1) multifocal small tongues or islands of viable cartilage extend into the primary spongiosa, (2) moderate-sized tongues or islands of viable cartilage extend into the primary and secondary spongiosa, and (3) extensive areas of the zone of hypertrophy are expanded and extend down into the primary and secondary spongiosa (Figure 4).

### 2.6. PRRSV Immunohistochemistry (IHC)

Paraffin-embedded tissue of the 10th rib was sectioned at 4 μm and placed on coated slides prior to immunohistochemical staining. The slides were submitted to the North Carolina Veterinary Diagnostic Laboratory System (Rollins Laboratory) for the immunohistochemical assay. The PRRSV IHC was conducted on the Leica Bond Max using a cocktail of mouse monoclonal antibodies SDOW17 and SR30 (Rural Technologies, Inc., Brookings, SD, USA) by diluting 1 μL of each antibody to 1500 μL of ultra vision serum-free protein block. The enzyme epitope retrieval method was used for 10 min, followed by 15 min of primary antibody, and power-vision anti-mouse AP secondary antibody for 8 min. The slides were then counterstained with hematoxylin.

### 2.7. In Situ Hybridization of PRRSV and CD163

Colocalization of PRRSV and CD163-positive cells in the bone was performed with RNAscope™ in situ hybridization (ACDBio, Newark, CA, USA). Briefly, decalcified 4 µm sections of bone were placed on positively charged slides. Formalin-fixed paraffin-embedded sample prep and pretreatment for the ACD RNAScope 2.5 assay (Document #322452) was modified for pretreatment of slides by using 100 µL of pretreatment fluid per slide. For antigen retrieval and protease treatment of bone, the standard condition was determined to be optimal with 15 min of antigen retrieval followed by 30 min of incubation with protease. For chromogenic staining of slides, the RNAScope 2.5 HD duplex detection kit was used with adherence to the corresponding protocol (Document #322500-USM). The following ACD probes were used: Ss-CD163 (#530501) and V-PRRSV-ORF1a-PRRSV-2 isolate USA/MN/01775GA/2021 (#1589061-C2). Of note, Amps 5, 6, 9, and 10 were incubated on slides at room temperature. For counterstaining, slides were incubated in hematoxylin for 30 s, followed by one wash in water for 30 s. Slides were digitally scanned with a RNAscope™ in situ hybridization (ACDBio, Newark, CA, USA). at 20× objective magnification.

### 2.8. Statistical Analysis

Statistical analysis was performed in GraphPad Prism version 11.0.0 (GraphPad, La Jolla, CA, USA). The pig survival rate was assessed using the Kaplan and Meier product limit method and compared using the log-rank test. The differences between parameters were assessed among the treatment groups using one-way or two-way ANOVA followed by multiple comparison analyses. A statistical threshold of *p* < 0.05 was used as a cutoff.

## 3. Results

### 3.1. Changes in the 25-OH D3, PTH, Calcium, and Phosphorus Blood Levels

The average serum 25-OH D3 levels from day −19 to 14 DPC in the Supplemented group (7.79–49.80 ng/mL) were significantly higher (*p* < 0.05) than all other treatment groups (Control (3.63–9.7 ng/mL), Marginal (1.83–4.52 ng/mL), Standard (3.04–11.28 ng/mL) throughout the study (Figure 1, Supplementary Table S2). At 7 and 14 DPC, the serum calcium and phosphorus levels decreased in all PRRSV-infected groups, with significant differences in serum calcium (*p* < 0.05) levels observed at 7 DPC between the Supplemented and Standard group when compared to the Control (Figure 1). The serum calcium level of the Marginal group at 7 DPC was the lowest. However, no significant differences were noted when compared to the Control due to the high degree of data variation. The average ionized calcium level at −5 and 0 DPC in all groups was within 1.35–1.44 mmol/L. At 7 DPC, the ionized calcium in PRRSV-infected pigs dropped to the range of 1.21–1.24 mmol/L, then increased back up to 1.38–1.45 mmol/L by 14 DPC. Likely in response to the decreased ionized calcium at 7 DPC, the average PTH levels in PRRSV-infected pigs increased to the range of 4.49–5.57 pmol/L, while the Control was at 2.82 pmol/L (Figure 2, Supplementary Table S3).

### 3.2. Clinical Parameters and Mortality Related to PRRSV Infection

From 0 to 14 DPC, the average daily feed intake (ADFI) and average daily gain (ADG) in PRRSV-infected pigs were significantly lower than those of the Control group (Table 1). All challenged pigs had gross and histopathological changes consistent with PRRSV infection, characterized by interstitial pneumonia. Additionally, PRRSV was detected in the serum via real-time quantitative polymerase chain reaction (RT-qPCR) at 7 and 14 DPC, confirming infection (Figure 3). At 7 and 9 DPC, pigs from the inoculated groups had significantly elevated body temperature with fever above 40 °C. The average body temperature remained high and eventually dropped within a normal range at 14 DPC in all PRRSV-infected groups (Figure 3). A total of four pigs were found dead at 4 DPC (Marginal group, n = 1), 9 DPC (Standard group, n = 2), and 11 DPC (Standard group, n = 1). Additionally, four pigs were euthanized due to either inability to rise without assistance, open-mouthed breathing, or neurologic signs at 9 DPC (Supplemented group, n = 1), 10 DPC (Marginal group, n = 2; Standard group, n = 1; Supplemented group, n = 1), and 11 DPC (Standard group, n = 1). Postmortem examinations were performed for all pigs by board-certified veterinary anatomic pathologists. The cause of death or severe decline was attributed to PRRSV systemic infection in all pigs that died or were euthanized during 9 to 11 DPC. The pig in the Marginal group that died acutely at 4 DPC was due to bacterial sepsis. The survival rates did not differ significantly between the dietary treatment groups (Figure 3). In the Control group, clinical signs and gross and microscopic lung changes were not observed, and all these pigs were PRRSV-negative (Ct > 40), as determined via RT-qPCR in the serum at 7 and 14 DPC.

### 3.3. Bone Phenotypic Changes Related to Vitamin D Levels and PRRSV Infection

The average physeal scoring of the 10th ribs in the Marginal group at 14 DPC (2.78 ± 0.44, mean ± SD) was the highest, followed by the Supplemented (2.33 ± 1.00), Standard (1.88 ± 1.25), and Control groups (1.67 ± 1.21) (Figure 4). However, no statistically significant differences were found with the physeal score, bone ash, and bone density between the treatment groups (*p* > 0.05). Of the eight pigs that succumbed to PRRSV infection prior to 14 DPC, five had lesions of myelonecrosis in the bone marrow (Figure 5). This lesion is characterized by multifocal aggregates of karyorrhectic and karyolytic hematopoietic precursor cells and areas around sinusoidal capillaries between the bone trabeculae. In situ hybridization (ISH) and immunohistochemistry highlighted PRRSV virus RNA and antigen scattered throughout the bone marrow and often concentrated in areas of necrosis (Figure 5). Viral RNA was identified within both CD163-expressing and non-expressing cells (Figure 5).

## 4. Discussion

In a conventional commercial production setting where pigs are housed indoors without access to sunlight, the majority of vitamin D is obtained through dietary sources. There are currently two forms of dietary supplements of vitamin D in pigs, including the traditional vitamin D3 (cholecalciferol) and 25-OH D3 (calcidiol), with the latter being considered to have higher bioavailability. The 25-OH D3 is a more polar and hydrophilic molecule with improved intestinal absorption. Its direct incorporation into the circulation bypasses the first hydroxylation step in the liver, a benefit that makes 25-OH D3 an ideal supplement in human patients with conditions such as liver disease or obesity [29]. Supplementation with 25-OH D3 has been shown to enhance sow reproductive performance, piglet growth performance, and significantly improve pig immune status [30]. Current swine production practices involve supplementing commercial swine diets with 5–11 times the minimum requirement for vitamin D set by the US National Research Council (NRC) [17,18,19,20]. The European Union Food Safety Authority imposes a strict cap on the amount of vitamin D in dietary supplements due to concerns regarding toxic levels [31]. Regardless, existing survey data reveal a discordance between vitamin D level in the feed and the measured serum 25-OH D3 status in the host [21,27,32]. A prior survey has shown low mean serum 25-OH D3 concentrations in nursery and grower pigs (2–14 weeks) at 11.4 ng/mL and 19.5 ng/mL (reference values 18–30 ng/mL), respectively [27]. Factors contributing to the low circulating vitamin D levels in post-weaning pigs include: low maternal transfer, housing indoors and the inability to synthesize sufficient amounts of vitamin D3 in the skin, poor intestinal absorption of ingested vitamin D3 (especially with low levels of dietary fat or poorly digestible fat), increased host utilization during an inflammatory condition, underlying liver insufficiency, and the inability to efficiently metabolize ingested vitamin D to circulating 25-OH D3 [27,33]. It is also important to consider the genetic and physiological processes underlying vitamin D metabolism. Specifically, different pig breeds exhibit variations in the gene expression of enzymes that convert vitamin D3 into its active form [34].

Our study observed that PRRSV-2 L1C.5 infection resulted in a decline in serum 25-OH D3, calcium, and phosphorus concentrations regardless of the level or form of vitamin D in the diet, suggesting either a direct effect of reduced nutrient intake or an increased cellular consumption of these nutrients during infection, particularly by the immune system. Notably, there is a significant decrease in serum calcium and a reduction in ionized calcium level with PTH response that is particularly evident at 7 DPC. This may indicate high host demands and utilization of calcium during this acute stage of PRRSV infection. These findings are aligned with observations in other viral infections. In severe acute respiratory syndrome coronavirus-2 and dengue fever virus, the reduction in the ionized calcium level is a poor prognostic marker resulting in higher disease severity in humans [15,16]. Moreover, some viruses exploit host calcium influx for entry and contain virulent factors (e.g., viroporin) that act as ion channels to facilitate survival [35]. The small envelope (E) protein in PRRSV demonstrated viroporin-like function that facilitates the uncoating of the virus during viral entry by opening the ion channel to allow calcium to move into the viral endosome and release the viral genomic material into the host’s cytoplasm [36]. Although serum calcium remained low in PRRSV-infected animals on 14 DPC, the difference was not statistically significant compared to the non-infected animals. This lack of significance is potentially due to limited statistical power resulting from animal attrition towards the end of the study.

Interestingly, supplementation of dietary vitamin D did not alter serum calcium, phosphorus, or ionized calcium concentrations during the first 14 days of PRRSV-2 L1C infection. Even with substantially higher serum 25-OH D3 in the Supplemented group, the evaluated biomarkers showed no significant differences. This lack of response is likely due to the tight regulation of converting 25-OH D3 into its active form [29]. Moreover, during the inflammatory process, the available vitamin D metabolites are redirected away from mineral regulation to support the immune function and cytokine production [30]. Finally, the short survival time and limited observation window may have constrained detectable changes in the biomarkers. An extended study with a larger sample size is required to determine whether dietary vitamin D influences the recovery of these biomarkers over time and to further explore its immune-mediated metabolic pathways.

In the present study, pigs infected with PRRSV-2 L1C.5 had reduced feed intake and growth, as well as elevated mortality, in accordance with previous reports for this strain [6,8]. Our study did not show significant differences in survival rate from PRRSV-2 L1C.5 infection between the dietary vitamin D treatment groups. While higher circulating vitamin D levels have been demonstrated to play a protective role in other swine diseases, such as porcine epidemic diarrhea and porcine proliferative enteropathy [37,38], this effect was not observed here. The lack of significant protection by vitamin D supplementation may be attributed to the high virulence of this strain and reduced feed intake, which may have overwhelmed the potential benefits of supplementation. Further research to determine whether vitamin D supplementation could alleviate the clinical disease of other, less virulent, and contemporary isolates of PRRSV should be explored.

Pathological changes in the bone with hypovitaminosis D are characterized by failure of endochondral ossification in growing animals (rickets) and fibrous osteodystrophy in adults [39,40]. Development of these changes requires time and prolonged vitamin D deficiency. Previous work has indicated that more consistent pathological physeal changes are observed with a phosphorus-deficient diet, though the most severe changes were observed with vitamin D deficiency [22]. The effects of different levels of dietary vitamin D on the bones of post-weaning pigs have not been fully explored, particularly in the context of PRRSV challenge. In our study, there were no significant differences in the physeal changes, bone ash, and bone density between the treatment groups. However, when comparing the PRRSV-infected pigs to the uninfected pigs, there was a trend of increased physeal changes related to failure of endochondral ossification in the PRRSV-infected pigs. Although dietary supplementation with vitamin D, particularly 25(OH) D3, during the 40-day study period resulted in a clear increase in serum vitamin D concentrations, the intervention period may have been insufficient in duration to induce significant pathological changes in the evaluated bones. A longer-term study would be necessary to fully explore the chronic effects of varying vitamin D status on bone health.

An interesting finding of this study was the microscopic changes in myelonecrosis in the bone marrow of pigs that died acutely or were euthanized due to severe clinical signs during the early stage of PRRSV-2 L1C.5 infection. Myelonecrosis was observed in 62.5% (5/8) of pigs starting at 9 to 11 DPC. This change was associated with PRRSV infection, as demonstrated by the localization of PRRSV antigen within the areas of necrosis and spreading throughout the intact hematopoietic cells in the bone marrow. The presence of PRRSV within the bone marrow has been reported for both PRRSV-1 and PRRSV-2. The changes in the bone marrow related to PRRSV infection are most notably characterized by hypoplasia of erythroid cells and an increased ratio of myeloid to erythroid precursor cells [11,12]. Furthermore, highly pathogenic PRRSV has been reported to induce apoptosis of the bone marrow cells from direct viral infection and as a bystander effect [10]. Differences in the degree of the bone marrow changes have been reported in different PRRSV strains and at specific time points post-infection [11]. The precise pathogenesis of the myelonecrosis observed in this study remains uncertain. It is possible that the changes in the bone marrow may be linked to a systemic vasculitis and the intense proinflammatory cytokine response that has been previously described with virulent PRRSV strains [41,42,43]. Additionally, we identified CD163-positive monocytes, the primary target cells for PRRSV, within the histological sections of the bone marrow. Colocalization of PRRSV and CD163 revealed viral nucleic acid within both CD163-expressing and non-expressing cells. This may be due to PRRSV infection through the use of alternative receptors, as has previously been proposed, the phagocytic uptake of viral genetic material by non-CD163 expressing cells, or a technical limitation of the dual chromogenic ISH assay in which red staining in infected cells overwhelmed teal staining for CD163 [44,45,46].

## 5. Conclusions

In conclusion, our findings demonstrate that infection by PRRSV L1C.5 causes high mortality regardless of the supplemental vitamin D levels. While supplementation successfully increased vitamin D levels, it did not alter serum calcium, phosphorus, or ionized calcium concentrations during the first 14 days of infection. We also observed a significant depletion of serum calcium during the early stages of PRRSV infection. Furthermore, this study identified novel microscopic changes of myelonecrosis in the bone marrow of PRRSV-infected pigs.

## Figures and Tables

**Figure 1:**
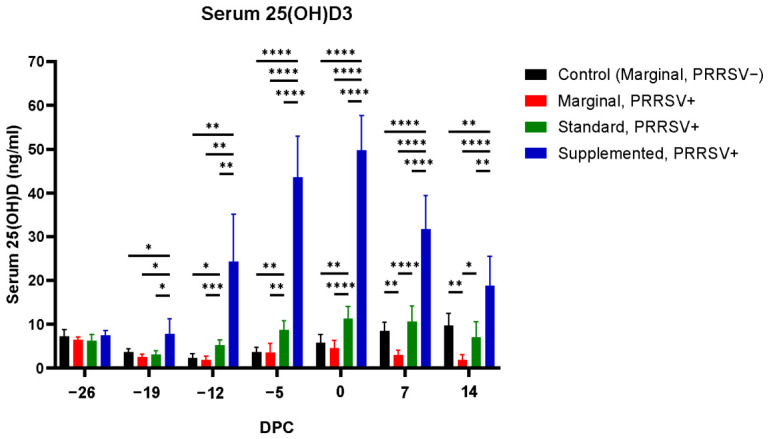
Average serum 25-hydroxyvitamin D3 levels of each treatment group at multiple time points throughout the study. Significance differences are indicated by asterisks (* *p* < 0.05; ** *p* < 0.01, *** *p* < 0.001; **** *p* < 0.0001) according to Tukey’s multiple comparison test.

**Figure 2:**
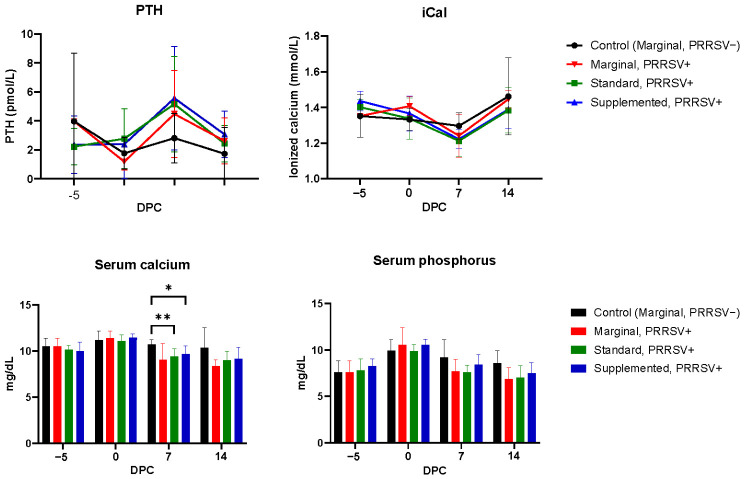
Average parathyroid hormone (pmol/L) ± standard deviation (SD), blood ionized calcium (mmol/L) ± SD, serum calcium (mg/dL) ± standard error of mean (SEM), and phosphorus (mg/dL) ± SEM levels in non-infected (Control) and infected pigs supplemented with vitamin D at Marginal (200 IU/kg), Standard (1500 IU/kg diet), and Supplemented (Standard + 2000 IU/kg of 25-hydroxy D3) levels. Significance differences are indicated by asterisks (* *p* < 0.05; ** *p* < 0.01) according to Tukey’s multiple comparison test.

**Figure 3:**
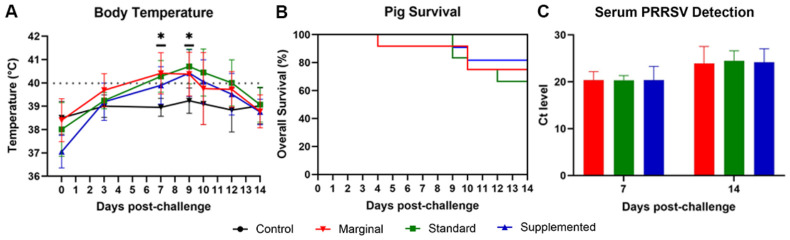
Clinical parameters and PRRSV detection in non-infected (Control) and infected pigs supplemented with vitamin D at Marginal (200 IU/kg), Standard (1500 IU/kg diet), and Supplemented (Standard + 2000 IU/kg of 25-hydroxy D3) levels. (**A**) Average body temperature (C°) ± standard deviation (SD) of pigs from each treatment group measured at 0, 3, 7, 9, 10, 12, and 14 DPC. The average temperature was above 40 °C (dotted line) during 7 to 10 days post-challenge (DPC). Compared to the non-infected Control, significant body temperature elevations occurred at 7 and 9 DPC for the Marginal (*p* < 0.01 and *p* < 0.05, respectively), Standard (*p* < 0.001 and *p* < 0.01, respectively), and Supplemented (*p* < 0.05 for both days) groups. Significance differences are indicated by asterisks (*) according to Tukey’s multiple comparison test. (**B**) Severe clinical disease that led to euthanasia and sudden death due to PRRSV infection started at 9 days DPC. At 4 DPC, one pig from the Marginal group died of bacterial sepsis. (**C**) The average level of cycle threshold (Ct) ± SD is displayed in the diagram for each treatment group. PRRSV was detected by RT-qPCR at 7 and 14 days in all infected pigs. The Ct cutoff was set at 40. The Ct level of all pigs in the Control group was >40 at both 7 and 14 DPC.

**Figure 4:**
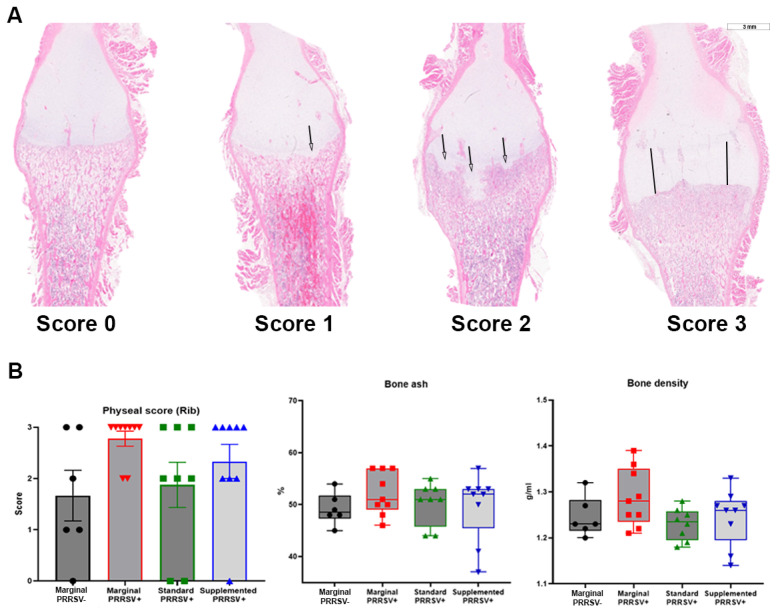
Bone morphological analysis results in non-infected (Control) and infected pigs supplemented with vitamin D at Marginal (200 IU/kg), Standard (1500 IU/kg diet), and Supplemented (Standard + 2000 IU/kg of 25-hydroxy D3) levels. (**A**) Histological physeal grading of 10th rib. A—Score 0 represents normal physis, B—Score 1 represents mild degree of failure of endochondral ossification characterized by multifocal small tongues or islands of viable cartilage that extend into the primary spongiosa (arrow), C—Score 2 represents moderate degree of failure of endochondral characterized by moderate-sized tongues or islands of viable cartilage that extend into the primary and secondary spongiosa (arrows), D—Score 3 represent rickets changes characterized by diffuse expansion of zone of hypertrophy that extend down into the primary and secondary spongiosa. (**B**) The physeal score of the 10th rib and femur, bone ash percentage and bone density (g/mL) of each treatment group.

**Figure 5:**
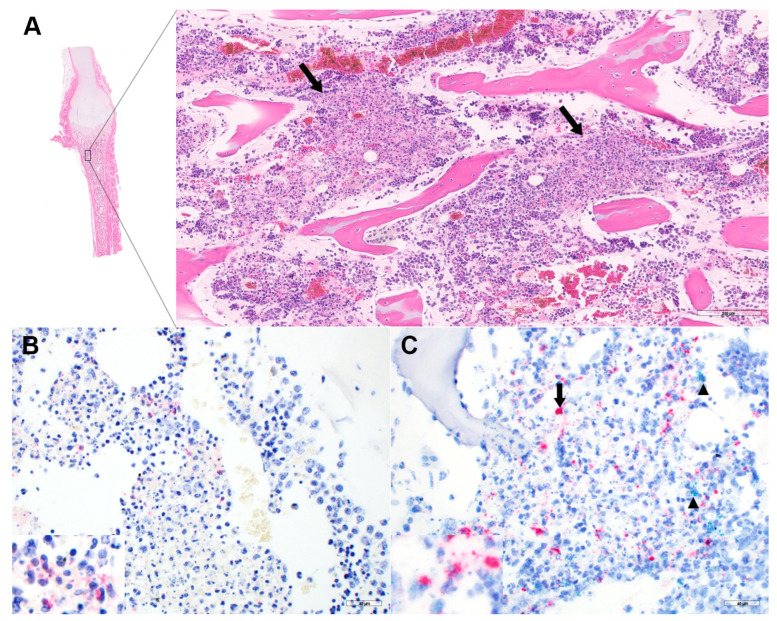
Histological images of the bone from a pig with sudden death at 11 DPC caused by systemic PRRSV infection. (**A**) 10th rib, H&E staining: There are multifocal areas of myelonecrosis distributed throughout the bone marrow spaces in the metaphysis and diaphysis (arrows). The necrotic areas are characterized by hypereosinophilic aggregates of degenerated and necrotic hematopoietic precursor cells that form clumps of karyorrhectic and karyolytic debris. (**B**) 10th rib, PRRSV IHC: There are scattered PRRSV immunopositive cells (red) within the areas of myelonecrosis. Hematoxylin counterstain. (**C**) 10th rib, PRRSV and CD163 multiplex ISH: PRRSV virus RNA (red, arrow) is found scattered throughout the hematopoietic cells in the bone marrow and concentrated in the areas of necrosis. Colocalization revealed PRRSV in CD163-positive (teal, arrowheads) and CD163-negative cells (inset). Hematoxylin counterstain.

**Table 1:** ADFI and ADG from 0 to 14 DPC.

Parameters	Control, PRRSV−	Marginal, PRRSV+	Standard, PRRSV+	Supplemented PRRSV+	*p*-Value
ADFI, kg/d	1.041 ^a^ ± 0.419	0.454 ^b^ ± 0.041	0.425 ^b^ ± 0.034	0.489 ^b^ ± 0.049	0.025
ADG, kg/d	0.658 ^a^ ± 0.071	0.093 ^b^ ± 0.043	0.084 ^b^ ± 0.068	0.104 ^b^ ± 0.046	<0.001

^ab^ Indicate significant differences at *p* < 0.05 according to Tukey’s multiple comparisons test; ADFI: average daily feed intake, ADG: average daily weight gain.

## Data Availability

The datasets used and/or analyzed during the current study are available from the corresponding author on reasonable request.

## Supplementary Materials

The following supporting information can be downloaded at: https://www.mdpi.com/article/10.3390/v18070711/s1, Table S1: Phase 1 and 2 diet compositions; Table S2: Average serum 25-hydroxyvitamin D3 levels of each treatment group at multiple time points throughout the study; Table S3: The mean and SEM of parathyroid hormone, blood ionized calcium, total serum calcium and serum phosphorus across study time points.

## Abbreviations

The following abbreviations are used in this manuscript:

25-OH D3	25-hydroxy-vitamin D3
ADFI	Average daily feed intake
ADG	Average daily gain
DPC	Days post challenge
IHC	Immunohistochemistry
PRRSV	Porcine reproductive and respiratory syndrome virus
RT-qPCR	Quantitative reverse transcription polymerase chain reaction
