# Supplementary material for: The Effect of Highly Virulent PRRSV-2 (L1C.5) Infection on Calcium Homeostasis and Bone Changes in Pigs Fed with Various Levels of Dietary Vitamin D
Source: Viruses. 2026 Jun 27;18(7):711. doi: 10.3390/v18070711 (PMC13431496; doi:10.3390/v18070711)
Supplement: Supplementary file 1 [file viruses-18-00711-s001.zip › viruses-4396043-supplementary.pdf]

Supplementary Table S1: Phase 1 and 2 diet compositions

**Ingredient**	**Phase 1**	**Phase 2**
Corn, 7.5% CP	50.56	64.69
Soybean meal, 47% CP	22.00	30.23
Whey permeate	12.50	0.00
Processed soybean product[1]	7.50	0.00
Fish meal, menhaden	3.50	0.00
Poultry fat	1.00	1.00
L-lysine HCl	0.45	0.47
DL-methionine	0.24	0.20
L-threonine, 80%	0.30	0.28
L-tryptophan	0.06	0.04
L-valine	0.09	0.09
Monocalcium phosphate, 21% P	0.67	1.31
Limestone	0.62	0.93
Salt	0.25	0.50
Experimental vitamin premix[2]	0.06	0.06
Trace mineral premix[3]	0.15	0.15
Colored grits[4]	0.05	0.05
**Calculated composition**		
Net Energy, kcal/kg	2.47	2.47
Crude Protein %	21.89	19.93
Lactose %	10.00	0.00
Total Lysine %	1.56	1.42
Calcium %	0.75	0.70
Phosphorus, %	0.68	0.67
Available Phosphorus, %	0.40	0.35

[1] HP300, Hamlet Protein

[2] Three separate vitamin premixes were used, containing 200 IU/kg and 1,500 IU/kg of vitamin D_3 for the marginal and industry treatment diets, respectively, and 1,500 ID/kg of vitamin D_3 plus 2,000 IU/kg of 25(OH) vitamin D_3 for the 25-OH D3 treatment diet. All other supplemental vitamins were identical between the premixes

[3] The trace mineral premix provided 33 mg/kg of manganese, 110 mg/kg of zinc, 110 mg/kg of iron, 17 mg/kg of copper, 0.30 mg/kg of iodine, and 0.30 mg/kg of selenium in the complete diet

[4] Colored grits were used to visually confirm the treatment diets

Supplementary Table S2: Average serum 25-hydroxyvitamin D3 levels of each treatment group at multiple time points throughout the study.

DPC	Control, PRRSV-			Marginal, PRRSV+			Standard, PRRSV+			Supplemented, PRRSV+		
	**Mean**	**SEM**	**N**	**Mean**	**SEM**	**N**	**Mean**	**SEM**	**N**	**Mean**	**SEM**	**N**
-26	7.28	0.74	4	6.39	0.26	8	6.26	0.48	8	7.50	0.39	8
-19	3.63[b]	0.38	4	2.48[b]	0.25	8	3.04[b]	0.32	8	7.79[a]	1.22	8
-12	2.33[c]	0.47	4	1.90[c]	0.29	8	5.19[b]	0.46	8	24.24[a]	3.89	8
-5	3.60[c]	0.58	4	3.49[c]	0.75	8	8.64[b]	0.77	8	43.58[a]	3.33	8
0	5.72[c]	0.79	6	4.52[c]	0.52	12	11.28[b]	0.79	12	49.80[a]	2.27	12
7	8.48[b]	0.83	6	2.93[c]	0.35	11	10.63[b]	1.03	12	31.64[a]	2.47	10
14	9.70[b]	1.13	6	1.82[c]	0.42	9	6.95[b]	1.27	8	18.81[a]	2.10	10

[abc] Indicate significant differences at $p < 0.05$ according to Tukey’s multiple comparisons test; DPC: Days post-challenge.

Supplementary Table S3: The mean and SEM of parathyroid hormone, blood ionized calcium, total serum calcium and serum phosphorus across study timepoints.

**Parameters**	**DPC**	**Control (Marginal, PRRSV-)**			**Marginal, PRRSV+**			**Standard, PRRSV+**			**Supplemented, PRRSV+**		
		**Mean**	**SEM**	**N**	**Mean**	**SEM**	**N**	**Mean**	**SEM**	**N**	**Mean**	**SEM**	**N**
PTH (pmol/L)	-5	3.98	2.10	5	3.98	2.10	5	2.23	0.63	4	2.36	0.89	5
	0	1.77	0.44	6	1.18	0.17	12	2.78	0.59	12	2.40	0.71	12
	7	2.82	0.70	6	4.49	0.91	11	5.17	0.95	12	5.57	1.13	10
	14	1.73	0.74	6	2.62	0.53	9	2.44	0.47	7	3.08	0.57	8
iCal (mmol/L)	-5	1.35	0.05	5	1.35	0.05	5	1.40	0.02	5	1.44	0.02	5
	0	1.33	0.03	5	1.41	0.02	10	1.34	0.03	12	1.37	0.03	12
	7	1.30	0.03	5	1.24	0.04	11	1.21	0.03	12	1.22	0.02	11
	14	1.46	0.09	6	1.45	0.02	8	1.38	0.05	8	1.39	0.04	9
Calcium (mg/dL)	-5	10.48	0.41	5	10.48	0.41	5	10.15	0.24	4	10.00	0.43	5
	0	11.18	0.41	6	11.41	0.22	12	11.08	0.20	12	11.47	0.11	12
	7	10.72[a]	0.21	6	9.06	0.52	11	9.39[b]	0.25	12	9.68[b]	0.27	10
	14	10.37	0.89	6	8.38	0.24	8	8.99	0.37	7	9.15	0.40	10
Phosphorus (mg/dL)	-5	7.60	0.54	5	7.60	0.54	5	7.80	0.61	4	8.24	0.36	5
	0	9.90	0.49	6	10.52	0.53	6	9.84	0.22	12	10.55	0.17	12
	7	9.20	0.78	6	7.69	0.38	11	7.55	0.23	12	8.42	0.34	10
	14	8.58	0.54	6	6.86	0.43	8	7.00	0.49	7	7.49	0.36	10

[ab] Indicate significant differences at $p < 0.05$ according to Tukey's multiple comparisons test; DPC: days post-challenge, SEM: standard error of mean
